# Supplementary material for: Attitudes and Approaches to Use of Meal Replacement Products among Healthcare Professionals in Management of Excess Weight
Source: Behav Sci (Basel). 2020 Sep 7;10(9):136. doi: 10.3390/bs10090136 (PMC7551264; doi:10.3390/bs10090136)
Supplement: Supplementary file 1 [file behavsci-10-00136-s001.pdf]

Attitudes and approaches to the use of meal replacement products amongst health professions in the management of excess weight

We are currently investigating the attitudes and approaches to the use and non-use of meal replacement products amongst health professionals working in weight management.

This survey will form the basis of a series of research investigations that aim to develop evidence-based practice in the use of meal replacement products.

As a health professional, you are the most qualified person to inform researchers on how you are managing your patients/clients with excess weight. We are looking for a range of responses from health professionals like you who work in weight management. We ask that you spend 10 minutes of your time to complete this survey. Your responses are valued and important to the future evidence-based practice of weight management.

We define meal replacement products as nutrient-complete food supplements. They are designed to replace meals and snacks in an energy controlled manner. These products can include, but are not limited to: shakes, soups, desserts or bars. Examples of common meal replacement brands that are currently on the market include: Optifast, Optislim, Proslim, Tony Ferguson, McCleods, Cambridge Diet and Kicstart. Note this is not an exhaustive list and other brands do exist.

Please note: not all questions will relate to your practice. Please tick “not applicable” where appropriate.

Demographic information and area of clinical practice (4 questions)

1. Please indicate your professional qualifications (select all that apply):

    - Endocrinologist
    - Cardiologist
    - General practitioner
    - Pulmonary physician
    - Gynaecologist
    - Oncologist
    - Orthopaedic surgeon
    - Bariatric surgeon
    - Nurse
    - Dietitian
    - Nutritionist
    - Exercise physiologist
    - Physiotherapist
    - Psychologist
    - Other (Please specify)

2. Please indicate the setting in which you work (select all that apply):
    - Hospital inpatient service
    - Hospital outpatient service
    - Private practice/rooms
    - Community health
    - Telehealth
    - Gymnasium
    - Other (Please specify)

3. What state do you work in:
    - NSW
    - ACT
    - QLD
    - WA
    - SA
    - TAS
    - NT
    - VIC

4. What type of location is your work place in:
    - Inner city
    - Urban area
    - Regional area
    - Remote area

Prescription patterns (15 questions)

5. Have you ever used meal replacement products to help your patients/clients manage excess weight?
    - Yes
    - No

5a. If you selected "No" What are the reasons you have not used meal replacement products as a weight management strategy for patients/clients with excess weight?

4. Have you ever had formal training on how to prescribe and use meal replacement products as a strategy for weight management? This may include university lectures, webinars, workshops or conferences.

    - Yes

- No

4a. If you have selected “Yes” please describe what training you have undertaken?

4b. If you have selected “No” how did you learn to prescribe and use meal replacement products for weight management? Please write N/A if this is not applicable.

5. What factors determine what type of patients/clients you prescribe meal replacement products for? (select all that apply)
    - BMI
    - Waist circumference
    - Fatty liver disease
    - Type 2 diabetes
    - Other obesity-related comorbidities
    - Age
    - Gender
    - Level of support available
    - Severity of obesity
    - Medications
    - Breathing difficulties
    - Weight loss required for surgery
    - Metabolic disease
    - Other (Please specify)
    - Not applicable

6. What percentage of your total patient/client load requiring weight loss is prescribed a diet containing meal replacement products? Please place an X on the line.

0% 25% 50% 75% 100%

7. How many days a week would you recommend a patient/client adhere to a meal replacement diet?
    - 1-2
    - 3-4
    - 4-5
    - 6-7
    - Not applicable

8. The rapid phase of a meal replacement diet is described as a time period which consists of the sole consumption of meal replacement products. It is often referred to as the intensive phase. Have you ever recommended clients undergo a rapid phase of a meal replacement diet to lose weight? Please exclude the preparatory phase before bariatric surgery.

   - Yes
   - No

   8a. If you have chosen "Yes" how do you typically prescribe meal replacement products during the rapid phase of a meal replacement weight loss program?

9. How many meal replacement products do you typically prescribe as a daily intake when the goal is rapid weight reduction?
   - ≤3
   - 4
   - 5
   - ≥6
   - It varies according to an individual's height and/or weight
   - Not applicable

   9a. If you have chosen "It varies according to an individual's height and/or weight", please expand on how you structure your prescription of meal replacement products and why?

10. How many weeks do you typically prescribe the rapid phase of a meal replacement diet for weight loss, excluding prior to bariatric surgery?
    - ≤3 weeks
    - 4-6 weeks
    - 7-9 weeks
    - 10-12 weeks
    - ≥ 13 weeks
    - Other
    - Not applicable

    10a. If you have chosen "Other" please describe how many weeks do you typically prescribe the rapid phase of a meal replacement diet for weight loss, excluding prior to bariatric surgery?

11. Do you ever allow additional items in a meal replacement diet as part of your prescription for weight loss? Please select all that apply and suggest any others you routinely recommend.

    - No additional items are added
    - Non-starchy vegetables such as spinach, broccoli and tomato, and salad or vegetable soup
    - Food-based protein (e.g. meat, fish, eggs, chicken, pork or tofu)
    - Whey or casein protein supplements
    - Soy, pea, hemp protein supplements
    - Fibre
    - Multivitamin
    - Oil or fat (e.g. butter)
    - Electrolytes
    - Broth
    - Omega-3 fatty acids
    - Medium chain triglycerides
    - Diet jelly
    - Diet soft drinks
    - Diet chewing gum
    - Other (Please specify)
    - Not applicable

Perceptions surrounding compliance, durability and safety of MRPs (6 questions)

12. Have you ever experienced patient non-compliance with diets involving meal replacement products?
    - Yes
    - No

    12a. If you have chosen "Yes" what reasons do you believe contribute to patient non-compliance?

13. What do you believe is the main reason why meal replacement diets result in weight loss?

14. What is your experience regarding the long term outcome of weight loss and weight maintenance when it is achieved with a meal replacement diet?

15. In general, what are your perceptions about the safety of meal replacement programs as a weight loss tool?

16. Is there any further information or comments you would like to make?

Thank you for participating in our research project. If you have any concerns, enquires or would like to be updated on the outcomes of this survey please contact Gabrielle Maston: gabrielle.maston@nsw.health.gov.au
